# Complete Genome Sequence of *Epizootic hemorrhagic disease virus* Serotype 6, Isolated from Florida White-Tailed Deer (Odocoileus virginianus)

**DOI:** 10.1128/genomeA.00160-18

**Published:** 2018-04-05

**Authors:** Mohammad Shamim Ahasan, Kuttichantran Subramaniam, John A. Lednicky, Julia C. Loeb, Katherine A. Sayler, Samantha M. Wisely, Thomas B. Waltzek

**Affiliations:** a Department of Infectious Diseases and Immunology, College of Veterinary Medicine, University of Florida, Gainesville, Florida, USA; b Faculty of Veterinary and Animal Sciences, Hajee Mohammad Danesh Science and Technology University, Dinajpur, Bangladesh; c Department of Environmental and Global Health, College of Public Health and Health Professions, University of Florida, Gainesville, Florida, USA; d Emerging Pathogens Institute, University of Florida, Gainesville, Florida, USA; e Department of Wildlife Ecology and Conservation, University of Florida, Gainesville, Florida, USA

## Abstract

Here, we report the complete genome sequence of *Epizootic hemorrhagic disease virus* (EHDV) serotype 6 (EHDV-6), isolated from a Florida white-tailed deer (Odocoileus virginianus) in 2016. To our knowledge, this is the first full genome sequence determined for an EHDV-6 isolate from Florida.

## GENOME ANNOUNCEMENT

*Epizootic hemorrhagic disease virus* (EHDV) is a causative agent of hemorrhagic disease of farmed and wild cervids, including white-tailed deer (1, 2). This epitheliotropic double-stranded RNA virus is a member of the genus Orbivirus within the family Reoviridae (3). Biting midges of the genus Culicoides are confirmed vectors of EHDV (4). Clinical cases of EHDV infection vary from peracute death to a chronic disease course that includes respiratory distress, edema of the head and neck, oral erosions, and sloughing of the hooves (1). Ten EHDV serotypes (EHDV-1 through EHDV-8, 318, and Ibaraki virus) have been described (5), and of these, 7 (EHDV-1, EHDV-2, and EHDV-4 through EHDV-8) are globally distributed (6). Complete genome sequences of EHDV-1 and EHDV-2, isolated from Florida white-tailed deer, were recently reported (7). Although EHDV-6 has been reported in some parts of the United States (8, 9), the complete genome sequence of this serotype from white-tailed deer in Florida had not previously been determined.

A spleen sample was taken from a farmed white-tailed deer (animal no. OV208) suspected of having epizootic hemorrhagic disease (EHD) during a 2016 health assessment conducted by researchers from the University of Florida Cervidae Health Research Initiative. The presence of EHDV virus RNA (vRNA) in a homogenized spleen specimen was confirmed by reverse transcription-PCR (RT-PCR) (10) and followed by observation of virus-induced cytopathic effect (CPE) in Vero E6 cells (Cercopithecus aethiops [African green monkey] kidney, ATCC CRL-1586) 20 days postinoculation. The vRNA was then extracted from virions in spent medium using a QIAamp viral RNA minikit (Qiagen). A cDNA library was constructed using a NEBNext Ultra RNA library prep kit and sequenced on an Illumina MiSeq instrument. *De novo* assembly of the paired-end reads was performed in SPAdes v3.10.0 with the default parameters (11). All 10 EHDV segments were recovered from the Florida white-tailed deer isolate OV208. BLASTN analysis of the outer-coat protein (VP2) from isolate OV208 revealed the highest nucleotide identity (99%; 2890/2907) to an EHDV-6 isolate from the spleen of a white-tailed deer in Ohio in 2012 (12). The OV208 VP2 sequence was aligned in MAFFT 5.8 (13) to 79 orthologous EHDV-1, EHDV-2, and EHDV-6 nucleotide sequences retrieved from the GenBank database. A maximum likelihood (ML) analysis was performed in IQ-TREE (version 1.4.4) using 1,000 nonparametric bootstraps to test the robustness of the clades (14). Similar to the BLASTN results, the ML analysis supported placement of isolate OV208 as a member of the EHDV-6 clade most closely related to an EHDV-6 isolate from a white-tailed deer in Ohio (12).

The current study is the first to report the complete genome sequence of an EHDV-6 isolate from Florida white-tailed deer. A recent investigation (7) and the present study have sought to characterize the EHDV serotypes circulating in Florida white-tailed deer as the first step in the development of an efficacious vaccine to curb the impact of EHD on farmed deer populations.

### Accession number(s).

The genome sequence for EHDV-6, isolated from a Florida white-tailed deer (animal no. OV208), has been deposited in GenBank under the accession no. MG886400 to MG886409.
